# Supplementary material for: Dynamic molecular changes during the first week of human life follow a robust developmental trajectory
Source: Nat Commun. 2019 Mar 12;10:1092. doi: 10.1038/s41467-019-08794-x (PMC6414553; doi:10.1038/s41467-019-08794-x)
Supplement: Supplementary file 1 — Supplementary Information [file 41467_2019_8794_MOESM1_ESM.docx]

**Dynamic molecular changes during the first week of human life follow a robust developmental trajectory**

# Supplementary Methods

## Blood Processing SOP

We collected 1 ml of whole blood into heparinized blood collection tubes. A portion (200 µl) of the blood was immediately aliquoted into RNA-stabilizing buffer and then cryopreserved. After centrifugation of the remaining blood, plasma (~200 µl) was harvested and stored frozen in multiple aliquots for later analyses of soluble immune molecules (e.g. cytokines and chemokines), as well as plasma proteins and metabolites. The remaining cellular fraction (i.e. red and white blood cells (RBC; WBC) as well as platelets) was distributed into tubes pre-filled with RBC-lysis buffers and fixatives; after further wash steps these tubes were stored for later analysis of cellular subsets by flow cytometry (immune phenotyping in Figure 1). This retained a substantial portion (400 µl) of the cellular fraction enabling additional analyses (Other) as desired. We thus achieved our goal to utilize < 1 ml of newborn whole blood to generate the biological samples required for broad, systems-based analyses.

A total of 30 infants were enrolled in each of our cohort sites, The Gambia and Papua New Guinea (PNG), with 2 blood draws obtained per participant during the first week of life. The blood samples collected were processed on site and subdivided as per the SOP. The number of samples that were run in each OMICS platform varied depending on whether two blood samples of the same subject were obtained to enable longitudinal analysis (i.e., indexing), and the fulfillment of quality controls applied to ensure pristine data. As a result, 279 samples were run through the different OMICS platforms for the Gambia cohort of newborns (Supplementary Table 1) and 209 for the PNG cohort of newborns (Supplementary Figure 9).

## Immune Phenotyping across 1^st^ week of life

Given the increasing number of parameters assessed by e.g. flow cytometry (FCM), one of the four tubes prepared for FCM as described above (i.e. 100 μl) was sufficient to capture all of the pre-defined target cell populations, including low abundance cells (< 0.5% of total white blood cell count) such as plasmacytoid dendritic cells (pDCs) at sufficient numbers to permit robust cell population discovery (Supplementary Figure 2 as well as Supplementary Note 1).

## Transcriptomic analysis across 1^st^ week of life

Gene expression analysis by quantifying mRNA is common to most systems biology studies^1, 2^. Irrespective of the methodological platform used (e.g. microarray or RNA-Seq), the first step involves extraction of RNA of sufficient quantity and quality (e.g. as assessed by RNA integrity numbers^3^). We included adult whole blood samples as a technical control in our efforts to determine the experimental parameters necessary for successful adaptation of this method to small blood volumes from newborns. While 50-100 μl of newborn peripheral and cord blood would have sufficed to provide sufficient high-quality RNA for RNA-Seq (Supplementary Figure 3A and B), we chose to reserve 200 μl for RNA extraction since this allowed downstream application of various experimental quality control tests for optimization of the current SOP.

RNA-Seq analysis of our newborn peripheral blood samples generated the following total number of uniquely mapped reads: minimum of 2,630,269, maximum of 14,634,580 and median of 7,292,011 reads. Analysis of the whole blood data unsurprisingly revealed a large proportion of hemoglobin (Hb) transcripts; but many of these Hb sequences mapped to multiple rather than single Hb genes, specifically HBG1 (ENSG00000213934), HBG2 (ENSG00000196565), HBA1 (ENSG00000206172), and HBA2 (ENSG00000188536) (Supplementary Figure 3C). In addition, the overall proportion of Hb transcripts observed was far higher than what we typically observe in pediatric whole blood samples: for uniquely aligned reads, among the neonatal samples, we observed a mean of 60% and a median of 66% of reads aligning to Hb. In contrast, among samples from older children, we usually observe a mean of 26% and a median of 22% of reads uniquely aligning to some form of Hb. This suggests the presence of abundant splice variants of Hb genes in the first week of life^4^. The large number of Hb transcripts in whole blood can be dealt with by employing physical globin removal steps or increasing sequencing depth followed by bioinformatic removal. We previously had shown that bioinformatic removal provides more unbiased data as compared to physical removal^5^. After bioinformatically removing globin transcripts, the total numbers of uniquely mapped reads in our sample set were: minimum of 1,004,853, maximum of 8,628,721 and median of 2,648,631 reads. While male vs. female subjects could readily be distinguished in our cohort of neonates, sex accounted for only 6% of variance on PCA plots of the RNA-Seq data (Supplementary Figure 3G); we thus pooled data irrespective of sex for the remainder of our study.

We observed that genes with decreased expression as a function age were known to be involved with cellular responses to stress (adj. p-value = 8.3E-27 (DOL3 vs. DOL0), 9.7E-89 (DOL7 vs. DOL0)), detoxification of reactive oxygen (adj. p-value = 2.2E-06 (DOL3 vs. DOL0), 4.0E-31 (DOL7 vs. DOL0)), as well as heme biosynthesis (adj. p-value = 6.5E-35 (DOL3 vs. DOL0), 5.3E-28 (DOL7 vs. DOL0) and iron uptake (adj. p-value = 5.7E-11)(DOL3 only). Conversely genes involved in interferon signaling (adj. p-value = 1.5E-86 (DOL3 vs. DOL0), 1.9E-72 (DOL7 vs. DOL0)), negative regulation of RIGI (adj. p-value = 2.4E-31 (DOL3 vs. DOL0), 7.9E-10 (DOL7 vs. DOL0)) and complement activation (adj. p-value = 3.8E-19)(DOL7 only) were up-regulated with increasing age over the first week of life.

## Proteomic analysis across 1^st^ week of life

We found 10 differentially abundant plasma proteins at DOL1 vs. DOL0, 19 when contrasting DOL3 vs. DOL0, and 41 when contrasting DOL7 vs. DOL0 (Figure 3B) Analogous to the transcriptomic analysis, Hb protein was abundant in our samples. Hb can be released from RBC by hemolysis, which could occur for several reasons during plasma preparation. We reasoned that the degree of hemolysis could be sample specific. We thus correlated the pairwise plasma concentrations of Hb subunits -alpha (HBA1), -beta (HBB), -gamma-1 (HBG1), and -gamma-2 (HBG2) for each sample. This confirmed that the plasma concentrations of the different Hb subunits displayed statistically significant relationships to one another (Pearson correlation coefficient (R) > 0.5, t-test p-value<0.01), demonstrating that the ratio of the different Hb subunits were conserved in the first week of life (Supplementary Figure 4B, Supplementary Data 6).

At DOL3 vs. DOL0, three pathways were upregulated that center around the complement cascade. At DOL7 vs. DOL0, 5 pathways were upregulated including scavenging heme from plasma (hypergeometric distribution test adj. p-value = 9.5E-05), signaling to RAS (hypergeometric distribution test adj. p-value = 1.2E-03) and those already observed on DOL3. There were no downregulated pathways on DOL1 vs. DOL0.

## Metabolomic analysis across 1^st^ week of life

Metabolomic analysis is a rapidly evolving field^6^ with publicly accessible reference databases currently having limited curation relative to those available for transcriptomic or proteomic analysis^7^. We collaborated with Metabolon Inc. in our data acquisition of the plasma metabolome over the first week of life. We identified 202 differentially detected metabolites at DOL1 vs. DOL0, 457 at DOL3 vs. DOL0, and 508 at DOL7 vs. DOL0 after FDR correction (Figure 3C; Supplementary Figure 5). These differentially detected metabolites demonstrated enrichment in metabolic pathways after hypergeometric tests found on follow-up DOL compared to DOL0, namely aminoacyl-tRNA biosynthesis ((adj. p-value = 0.053, DOL1 vs. DOL0), 0.2 (DOL3 vs. DOL0), 0.02 (DOL7 vs. DOL0)), amino acid alanine, aspartate and glutamate metabolism (adj. p-value = 0.007 (DOL7 vs. DOL0)) and citrate acid TCA cycle (adj. p-value = 0.017 (DOL7 vs. DOL0)) suggesting rapid protein synthesis, cell proliferation and development of metabolism in newborns (Supplementary Data 7).

## Data integration

MOLECULAR INTERACTION NETWORKS: While each of the OMIC data types that we examined revealed statistically significant changes over the first week of life, we used as seed nodes only transcriptomic or metabolomic data to construct PPI-networks in NetworkAnalyst as they represented the two highest-powered OMICs data. Specifically, both transcriptomics and metabolomics were well powered for the PNG data set (80% power to detect age-dependent differences with n = 6); only the proteomic data was not. However, this was unlikely to have any impact on our integration analysis as the features used as inputs to NetworkAnalyst were strictly derived from the two well-powered OMICs data (i.e. transcriptomic and metabolomics). Furthermore, since lack of power increases the risk of Type II errors (false negatives), we would not expect the pathways identified from the proteomics data to be false positives; indeed, we note that in our integration studies we obtained good coherence between all three omics in terms of conclusions.

DIABLO: DIABLO constructs components – i.e. linear combinations of the underlying features – that that are maximally correlated across any number of input data types and a specified response variable (in this case, DOL), while simultaneously performing feature selection via L1 penalization^8^. This method is closely related to Projection to Latent Structures (PLS; sometimes called partial least-squares) regression, which has been successfully applied to the modeling of signaling networks measured by multiple technologies^9^. However, DIABLO extends this approach to integrate jointly multiple data matrices and identifies the main discriminant drivers in the data (schematically shown in Figure 5A). In addition, the connections between data blocks allows effective modeling of interconnections between the various data types. Specifically, matrices from our five data types (transcripts, proteins, metabolites, cytokines/chemokines, and cell composition) were used as input to DIABLO to identify the major combinations of ontogeny-related features amongst transcripts, proteins, metabolites, cytokines/chemokines and cell types in our datasets (Figure 5 and Supplementary Data 9). A model composed of 2 components and 100 features selected jointly across all 5 data types (2 x 10 features per data type) was fit based on the results of a cross-validation study (Supplementary Figure 6). This model discriminated well between DOLs.

To investigate the nature of the relationships between features selected by DIABLO across the different OMIC data types we used the model feature variates and loadings (analogous to eigenvectors in PCA) to reconstruct the data blocks and compute all pairwise correlations between features. The resulting correlation matrix was taken as an adjacency matrix to form a network, where nodes are correlated features selected by DIABLO and edges are Pearson’s correlation coefficient computed on the weighted values. After applying a quantile-based cut-off (top 25% of edges by weight using Pearson’s correlation coefficient), the selected features formed a network composed of 73 connected nodes and 963 connections (edges). (Since DIABLO allows features to be present in multiple components, 27 features were present in both the first and second component; however, duplicated features formed single nodes in the network.) Using an established topological algorithm ^10^, we partitioned the network into closely correlated subgraphs. The resulting 2 modules corresponded to the 2 components of the DIABLO model. We compared (Figure 5A vs. 5B) this integrative network to one derived from features identified using a non-integrative sparse discriminant analysis approach (sPLS-DA ^11^, applied separately to each data block). The integrative network was more densely connected (global clustering coefficient = 0.91 vs. 0.68) and composed of fewer (number of subgraphs = 2 vs. 41), tightly intra-, but loosely inter-correlated subgraphs, or modules (network modularity = 0.26 vs. 0.09), indicating that DIABLO selected groups of discriminant features that were well correlated across data blocks, while the non-integrative approach favored features that were discriminant, but not well correlated across data blocks. The features identified by DIABLO were largely distinct from those identified by the non-integrative method (Figure 5D; red bar) and, when we assessed them for pathway over-representation, corresponded better to well-understood biology than those identified by the non-integrative approach (77 vs. 27 Reactome pathways were significantly over-represented at adj. p-value < 0.10; Figure 5E).

Finally, we evaluated the ability of our model to generalize to artificially created test sets by carrying out repeated cross-validation (20x 5-fold). The lowest mean out-of-sample misclassification error rate (5.76 ± 2.76%) was achieved by the integrative DIABLO model. This performance was significantly better than that achieved by the best performing single data block model (mean error rate 8.73 ± 2.57% using the transcriptomic data alone; Wilcoxon rank sum test p-value = 0.002). The high accuracy suggests that the features identified by DIABLO were robustly associated with neonate ontogeny.

MMRN: In their study of the immune response to *Varicella zoster* vaccination, Li and others derived multi-scale, multi-response networks (MMRNs) following vaccination and identified “stable networks”, i.e. groups of features comprised of a single data type that co-cluster independently at many time points and associated across data types^1^. To construct MMRNs from our data, we first reduced dense data types, such as transcriptomic and metabolomic data, to modules and pathways. All data types were then clustered and significant associations between these clusters across data types identified. We derived MMRNs separately at each DOL and identified stable networks across DOL. Each node represented a cluster of one data type, with the edges representing significant associations between clusters across data types.

Using MMRN, we found that associations between data types were strongest at DOL1 and decreased across the first week of life, as partial least squares regression scores were significantly higher at DOL1 compared to all other time points (Student’s t-test, p-value << 0.01). Stable clusters were more strongly associated with DOL when compared to transient ones (Supplementary Figure 7C), with 15/21 clusters significantly associated with DOL being part of stable networks. This confirmed our already noted robust trajectory of development. Most of the significant clusters were transcriptomic (16/21), but we also identified metabolomic (1/21) and flow cytometry-derived (4/21) clusters associated with DOL (Supplementary Data 10).

Using this approach, we identified a stable B-cell cluster of cellular, transcriptomic, and metabolic networks that were consistently connected across all DOL and significantly associated with DOL (shown in Supplementary Figure 7D). These were associated with blood transcriptomic modules (BTMs) such as: enriched in activated dendritic cells/monocytes (M64), enriched in dendritic cells (M168), and TLR and inflammatory signaling (M16), but also, erythrocyte differentiation (M173), and heme biosynthesis (M171, M222), and, at DOL 3 and 7, BTMs including B-cell development (module M9), BCR signaling (M54), cell adhesion and migration, chemotaxis (M91), and cell-cell adhesion (M133.1) (but also included complement and other receptors (M40), enriched in activated dendritic cells (M165), activated dendritic cell surface signature (S11), inflammatory receptors and signaling (M53), and viral sensing and immunity, IRF2 targets network (M111.0)). These stable transcriptomic clusters were, in turn, associated with a stable metabolic pathway cluster that included the endocannabinoid pathway, both branched and monohydroxy fatty acid metabolism, glycine, serine and threonine metabolism, lysine metabolism, polyamine metabolism, and purine metabolism; several of these could represent novel aspects of ontogeny, while others (e.g. purine metabolism) confirmed previously identified important developmental changes ^12^. *MMRN*-based analysis of our data revealed a strong association between the transcriptomic and metabolomic compartments on DOL1 in transient node-networks that did not persist (i.e. they were not detectable in DOL3 and 7 samples); these transient signals related to metabolites originating from the liver or intestinal microbiota (note: plasma metabolites are mostly of liver or intestinal (microbiome) origin ^13^) that seem to impact changes in blood cell transcriptomics.

META-INTEGRATION: We assessed the significance of the observed convergence across all three analytical methods, NetworkAnalyst, DIABLO and MMRN, by bootstrapping (i.e. simulating random draws of the 635, 308, and 84 respectively-enriched Reactome pathways and assessing overlap amongst these 1 million times) and confirmed that the degree of consensus 34 pathways between the three different data integration approaches we found was extremely unlikely to occur by chance (p-value << 0.001; Supplementary Figure 8).

CROSS-COHORT VALIDATION: The first DIABLO component of our model that was validated across both cohorts (The Gambia; PNG) was composed of features consistent with interferon signaling (Figure 6E): the abundance of interferon gamma itself increased markedly by DOL1 before gradually decreasing over DOL3 and 7 (Luminex multiplex cytokine assay). The genes *GBP1* and *GBP4* are IFN-inducible GTPases and important determinants of cell autonomous immunity; they are functionally closely associated with interferon signaling^14^. In parallel to the increase in interferon signaling, immunoproteasome genes *PSMB9* and *PSME2* were strongly induced with increasing age (transcriptomics)^15^. Interferon gamma causes a dose-dependent increase in levels of IgM^16^ and, by DOL3, IGHM protein abundance was increasing (mass-spectrometry proteomics). Homoarginine, a required substrate for nitric oxide (NO) production (by nitric oxide synthase), which mediates cytostatic and cytotoxic activity of macrophages for antimicrobial defense^17^ gradually decreased over DOL1, 3, and 7 (metabolomics), suggesting increasing production of NO following activation of macrophages by interferon gamma ^18^ Concurrently, the relative proportion of a number of innate immune cells in the circulating blood also steadily increased (mDCs, non-classical monocytes, CD56+CD16- NK-like cells).

The second DIABLO component of our model that was validated across both cohorts had a distinct granulocytic-flavor (Figure 6F)^19^. Eotaxin (eosinophils) and interleukin 8 (or neutrophil chemotactic factor) are classic granulocyte chemokines. The expression of neutrophil azurophilic granule component transcripts (*AZU1*, *BPI*, the serine proteases *CTSG*, *ELANE*, and *PRTN3*, as well as the antimicrobial peptides *DEFA3* and *MPO*), but also neutrophil specific granule component lactoferrin (*LTF*), was down-regulated at DOL1 vs. DOL0, but highest by DOL7. Neutrophil counts decreased steadily over the same time interval, suggesting that the observed expression changes reflect functional changes in neutrophils, not simply changes in the composition of blood. The protein abundance of another specific granule component, cathelcidin (CAMP; by proteomics), peeked at DOL1 and then gradually decreased, possibly as a result negative feedback and protein-production lagging gene expression. Similarly, galectin-3 binding protein (*LGALS3BP*), a ligand of neutrophil receptor galectin-3, which promotes integrin-mediated cell adhesion and migration out of the circulation, was lowest at DOL1 and gradually increased over DOL3 and 7, while its gene expression was strongly up-regulated from DOL0 onwards. Lastly, the terminal protein components of the complement cascade increased steadily over the first week of life in both cohorts (C8 and C9; Figure 6G)^20^.

Overall, the observed pattern in both cohorts was consistent with a rapid upregulation of cell autonomous and innate immune function in newborns^21^.

Our sample-processing method did not require new technological breakthroughs or laboratory methods only available to a select few. Rather, it combined several sample- and time-sparing approaches into one feasible, field-ready SOP. All samples were generated in the low resource settings of The Gambia (West Africa) and Papua New Guinea (Australasia). Our experimental platform that generated the data shown in this manuscript required only 500 μl of peripheral whole blood, which was well below the 1 ml newborn target that we set for ourselves as the desirable volume.

A signal prominently regulated over the first week of life related to Hb, possibly because new Hb must be rapidly synthesized during the transition from fetal to postnatal Hb^4^. Other signals centered around iron metabolism that likely relate to the physiological drop in serum iron within hours of birth presumed to reduce the risk for neonatal infection^22, 23, 24, 25, 26^. At the same time, the increase in particular signatures associated with immune stimulation may be related to the rapidly increasing density and diversity of colonizing microbes following birth^27^, as amplified pathways included interferon signaling, platelet degranulation, chemokine receptor, interleukin signaling and dendritic cell maturation, all of which are activated in response to microbial exposure^28, 29, 30^.

Lastly, the observed profound changes in the first week of life stand in stark contrast to the adult scenario, for which relatively stable steady state cell populations, transcriptomes, proteins, cytokines and metabolites (or metabolic phenotypes) are maintained over weeks to months^1, 31, 32^.

# Supplementary Figures & Table

**Supplementary Table 1.** **Sample distribution for analysis.** A total of 60 blood samples from the Gambia cohort were processed according to the diagram in Figure 1. Only participants that provided two samples and samples that passed quality controls were subsequently run in each specific platform which led to the exclusion of 10 samples from transcriptomic analysis, 1 sample from flow cytometric analysis, and 6 samples from metabolomic and cytokine/chemokine analysis. In total 279 samples were processed.


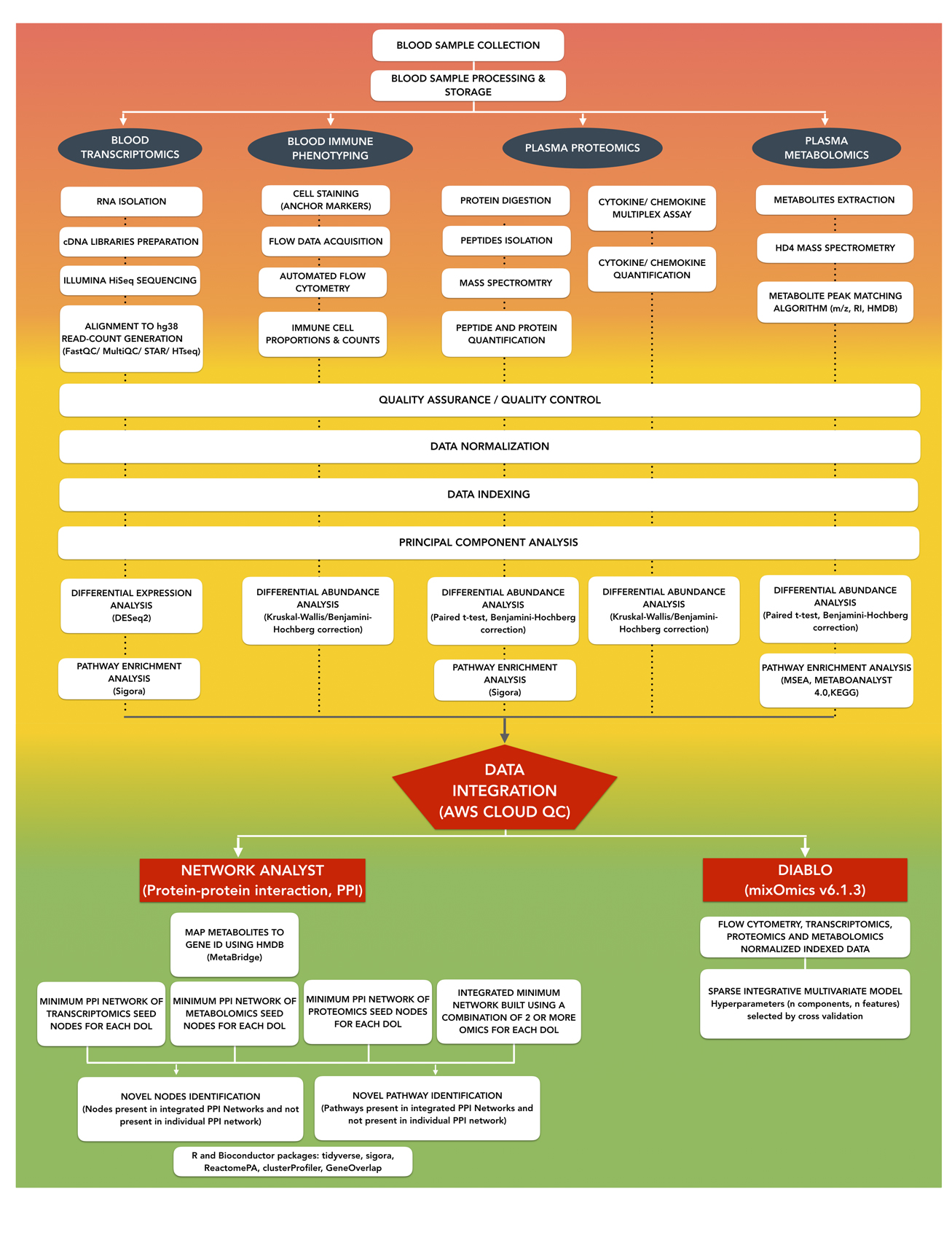


**Supplementary Figure 1. Work-flow diagram.** Sample processing steps as well as the statistical and bioinformatic analysis steps that were applied to the data are graphically summarized here to provide an overview. Details for each of the steps are provided in the Online Methods.


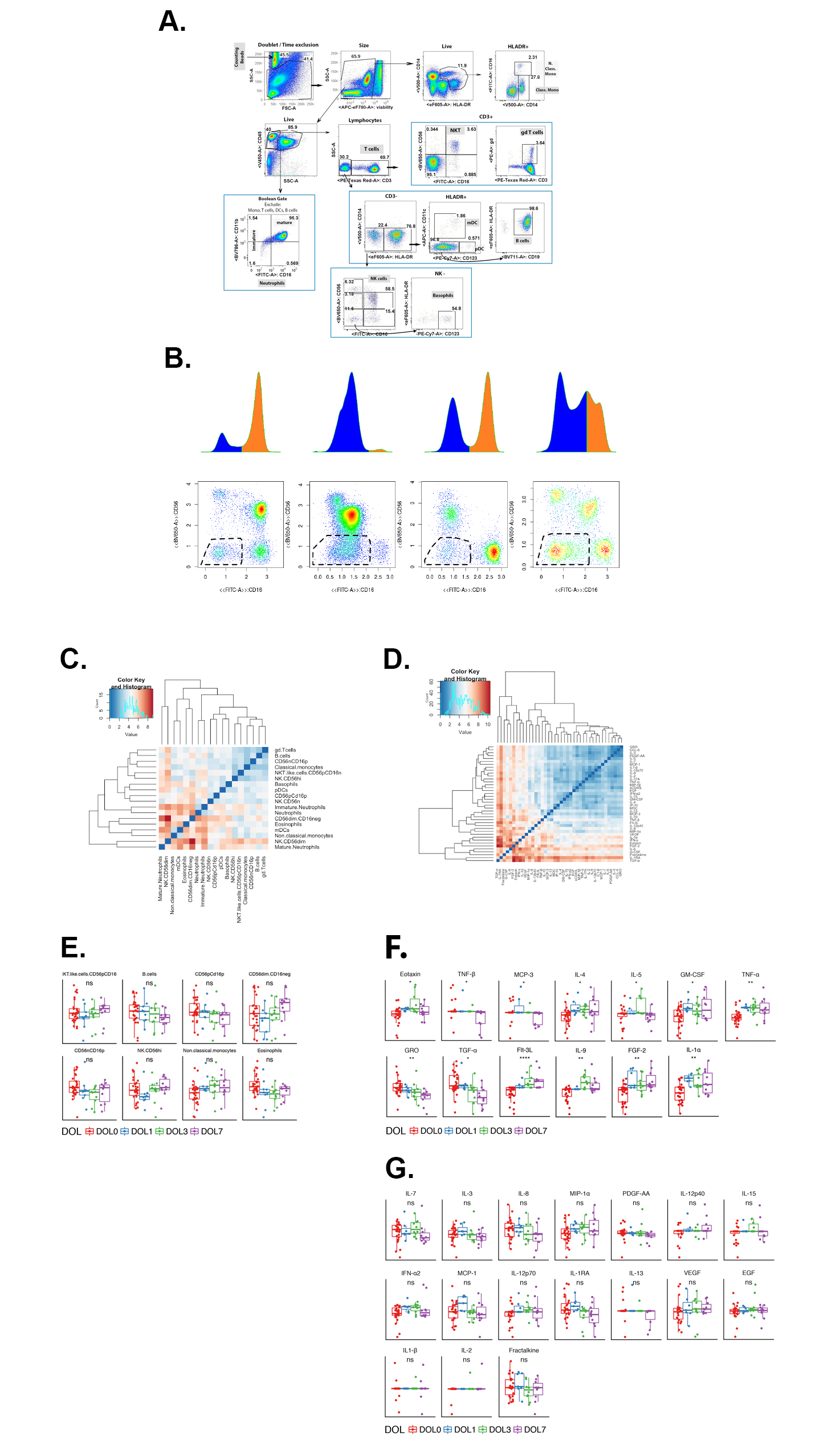


**Supplementary Figure 2. Flow cytometry and plasma cytokine and chemokine analysis. A.** Example of manual gating strategy **B.** Example of automated cell analysis of cell populations using flowDensity. Criteria for cell population thresholds were set in a supervised manner on a per-population basis based on density distributions to achieve robust, reproducible results. Gates were automatically adjusted for each FCS file in a data-driven manner, removing the subjectivity inherent in manual analysis. **C.** Inter-individual variability of cellular composition in whole blood was pronounced in early life. Heatmap showing the Euclidean distances between newborn DOL0 samples (blue indicates closely related samples while red indicates distantly related samples). **D.** Inter-individual variability in early life for plasma cytokine and chemokines. Heatmap showing the Euclidean distances between newborn DOL0 samples (blue indicates closely related samples while red indicates distantly related samples). **E**. Normalized cell counts and **F** normalized cytokine/ chemokine concentrations over the first week of life that displayed developmental trajectory; **G.** Normalized cytokine/ chemokine concentrations over the first week of life that did not display a change. Boxplots display medians with lower and upper hinges representing first and third quartiles. Whiskers reach the highest and lowest values, no more than 1.5 * interquartile range from the hinge **** p <= 0.0001, *** p <= 0.001, ** p <= 0.01, * p <= 0.05, ns p > 0.05, Kruskal-Wallis test, Benjamini-Hochberg adjusted p-values.


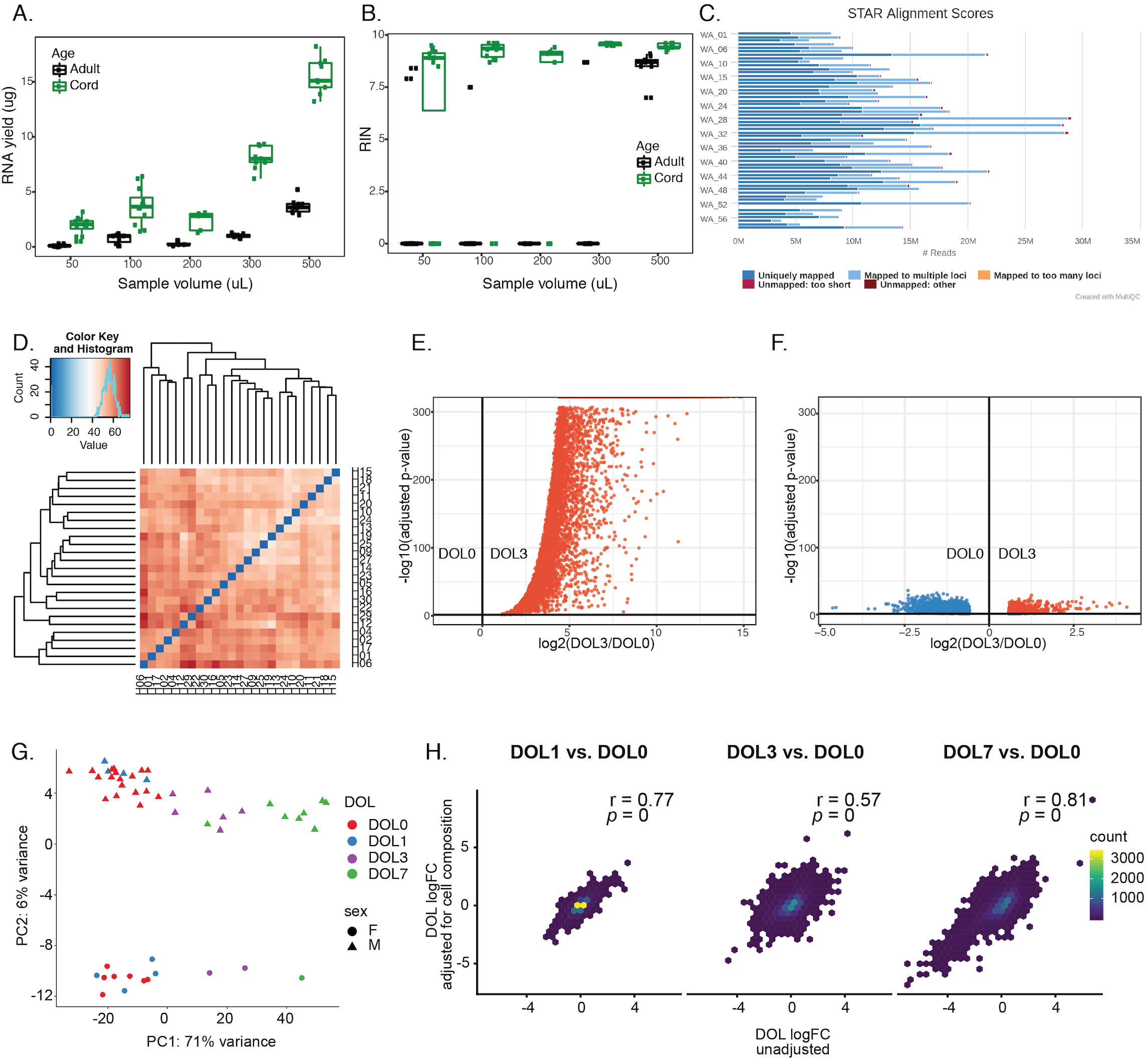


**Supplementary Figure 3. Transcriptomic analysis.** 50-100 μl of newborn cord blood suffice to consistently yield RNA of sufficient quantity (**A**) and quality (**B**) for subsequent RNA-Seq, while for adults > 500 μl were required. **C.** Hemoglobin genes dominated in whole blood obtained early life, especially in the multiple mapped reads category. **D.** Heatmap showing the Euclidean distances between newborn day of life 0 (DOL0) samples (blue indicates closely related samples while red indicates distantly related samples) demonstrated that inter-individual variability for transcriptome was pronounced in early life. **E and F.** Volcano plots generated from RNA-Seq data from whole blood after removal of globin gene transcripts *in silico*. Differential expression analysis was performed to identify differentially expressed (DE) genes between DOL3 and DOL0 using a model that either omitted indexing to DOL0 (E) or indexed to DOL0 (F). Of the 14,700 genes in the dataset, 3,029 were identified as being DE with the model in F that was indexed to DOL0, whereas virtually all expressed genes, 11,884, were indicated to be DE with the model in E, which would be biologically impossible and thus indicative of a poorly fitted model. **G.** Using normalized data for variance stability transformation, *in silico* globin gene removal, followed by indexing against DOL0 for each subject, PCA analysis from whole blood RNA-seq highlights the developmental trajectory along component 1. PCA plots of RNA-seq data from whole blood normalized by variance stabilizing transformation after bioinformatic removal of globin gene transcripts are shown for newborn data in panel **G**, where sex accounted for 6% of variance between samples. **H.** The cellular composition of the blood samples is not significantly confounding. The log2 fold changes from DESeq2 are compared for a simple model including only subject and DOL, and one including cell proportions as additional covariates. The log2 fold changes for both models were highly correlated, suggesting that variation in cell composition could not account for most of the observed variation in the transcriptomic data.


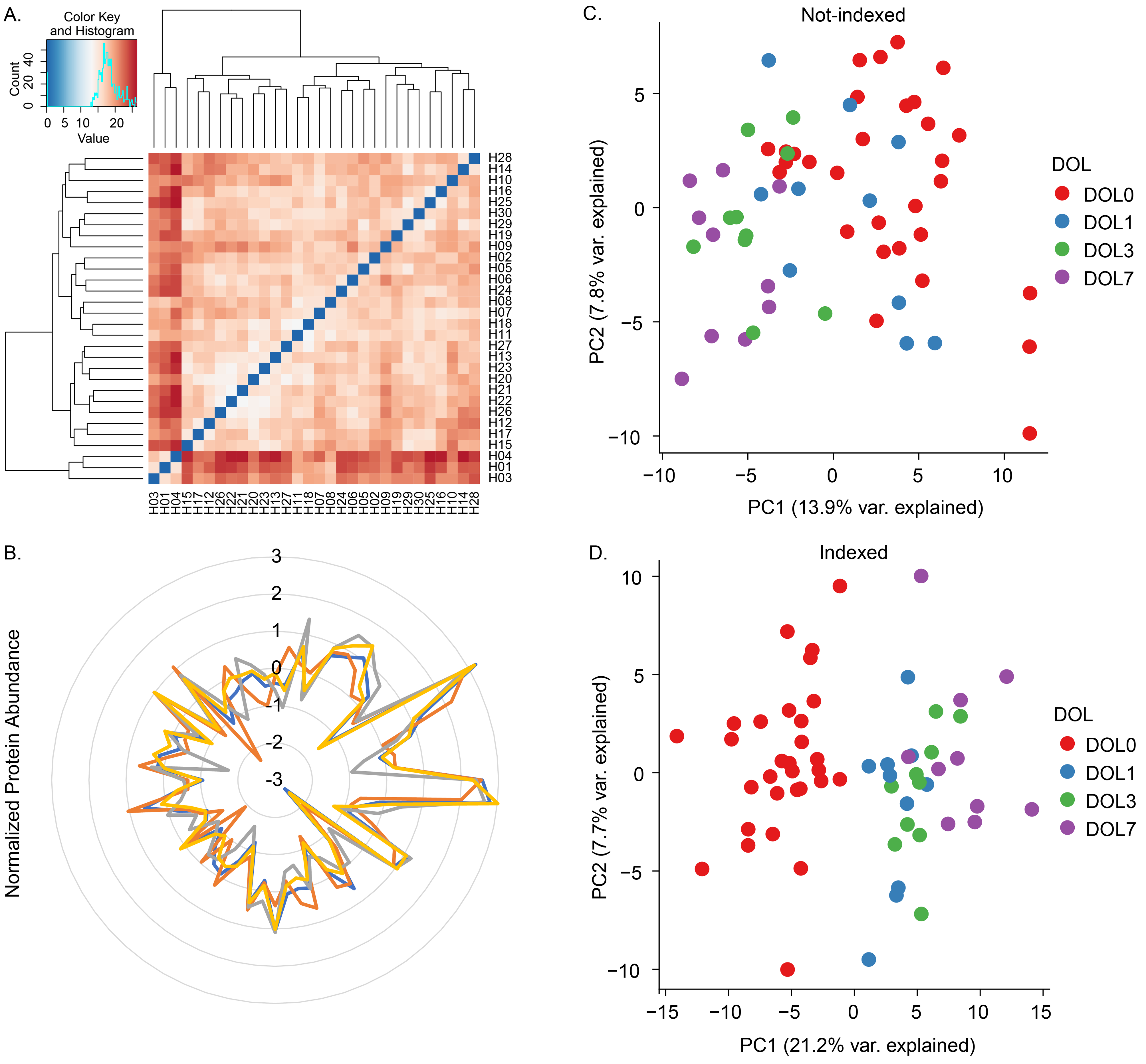


**Supplementary Figure 4. Proteomic analysis. A.** Heatmap showing the Euclidean distances between newborn day of life 0 (DOL0) samples (blue indicates closely related samples while red indicates distantly related samples) identified that inter-individual variability for plasma proteomics was pronounced in early life. **B.** Radar plot of the normalized hemoglobin subunit alpha (*HBA1*), hemoglobin subunit beta (*HBB*), hemoglobin subunit gamma-1 (*HBG1*), and hemoglobin subunit gamma-2 (*HBG2*) protein plasma concentrations across all 60 samples. A significant correlation (Pearson correlation coefficients (R) > 0.5, t-test p-value < 0.01) was found between the hemoglobin plasma abundances. This indicated that plasma hemoglobin contamination was sample specific, but the intrasample ratios of the various hemoglobin proteins were stable. **C.** Unsupervised principle component analysis (PCA) scores plot before and **D.** after accounting for repeat measures from the same individual across different sampling days compared to DOL0 (indexing to DOL0). Sample clustering by DOL between samples was seen along principle components (PC) 1 and 2, with indexing greatly improving the separation as a function of age.


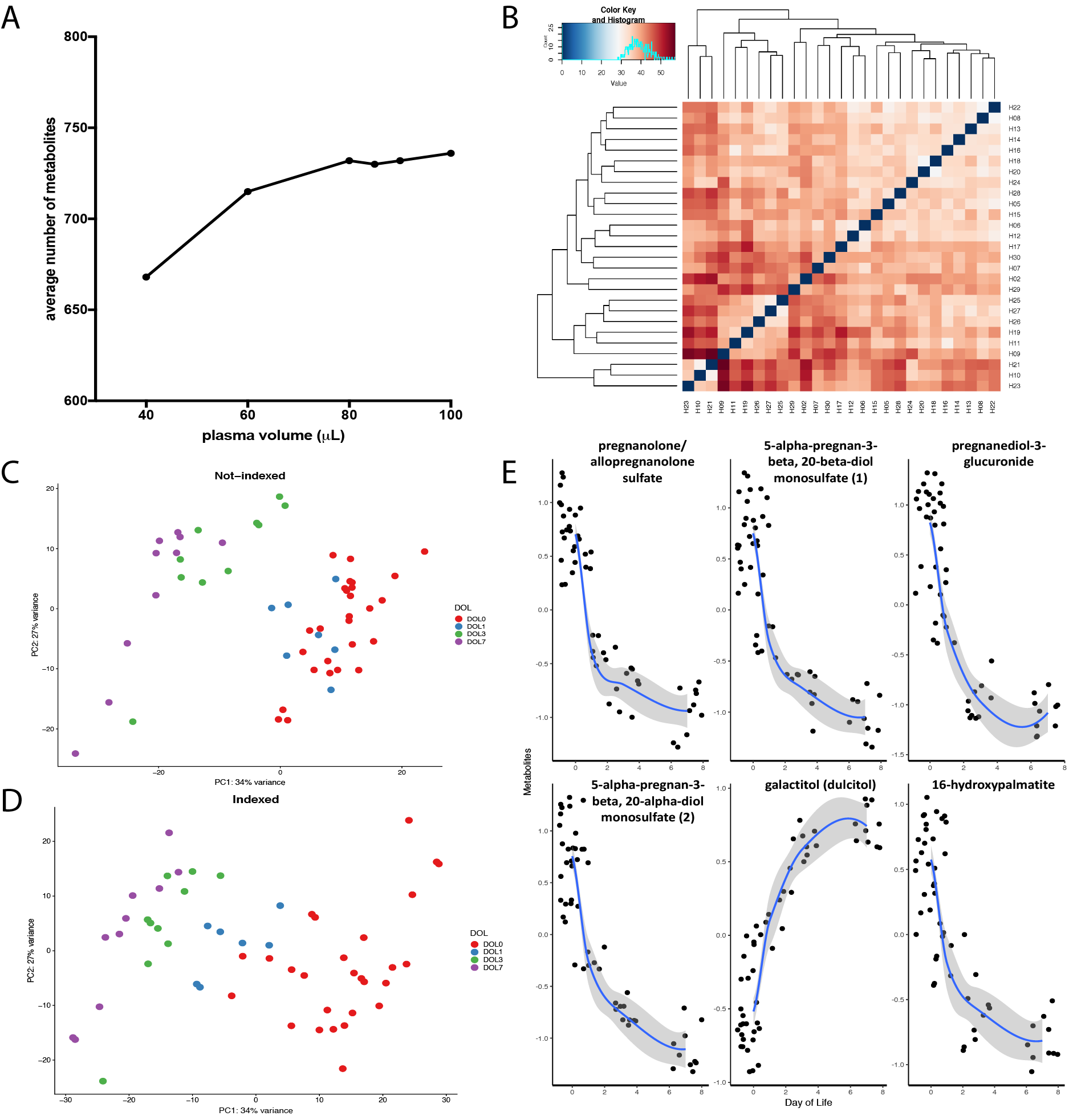


**Supplementary Figure 5. Metabolomic analysis. A.** Number of detectable metabolites per plasma volume. **B.** Heatmap of metabolomic features for each sample showing the Euclidean distances between newborn DOL0. **C**. PCA plot of unindexed metabolomics samples displaying the pronounced variation between subjects. **D.** Indexing, i.e. accounting for repeat measures from the same individual across different sampling days compared to DOL0 (indexing to DOL0) revealed clustering as a function of age. **E**. Analysis of steroids, a lipid subgroup, revealed a decrease in plasma of the first week of life and an increase in galactitol, a carbohydrate metabolite, displays an increasing trend over the first week of life.


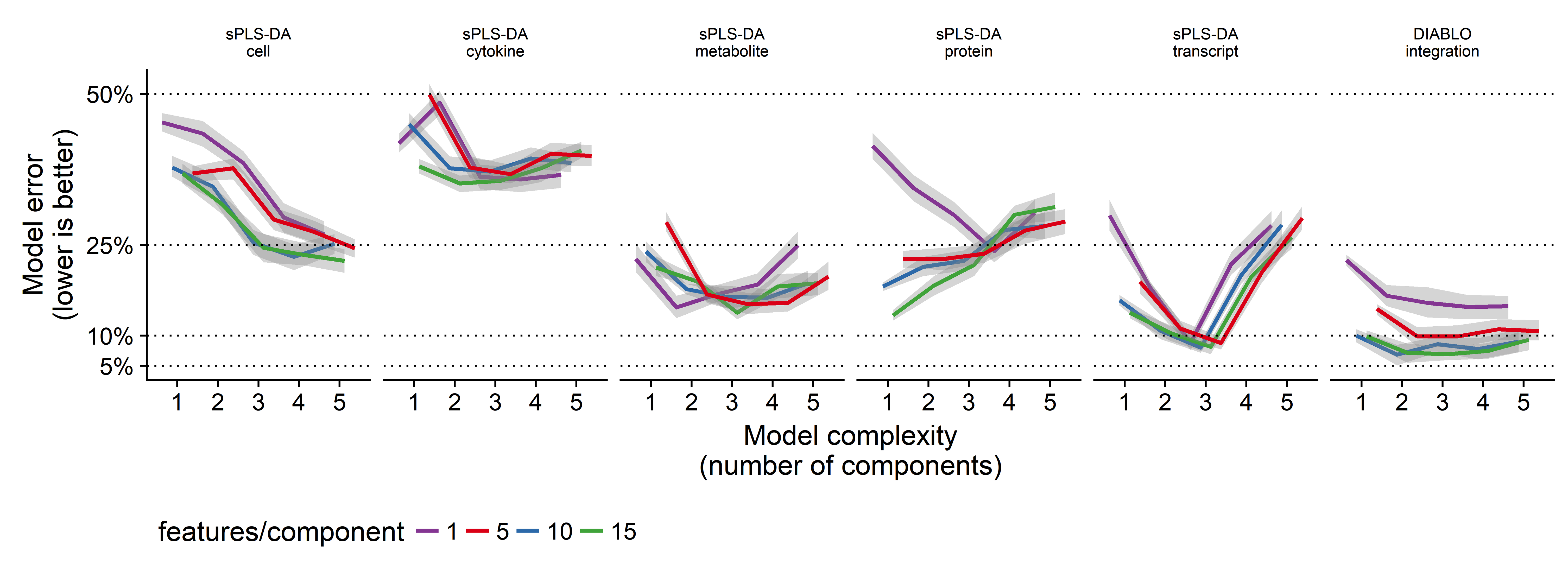


**Supplementary Figure 6. DIABLO cross-validation analysis.** We explored the relationship between overall model complexity (number of components; x-axis) and model generalizability to unseen data (mean error rate (and standard error; shadowed area) when predicting DOL in unseen samples; y-axis). Results of a repeated (20x) 5-fold cross-validation study are shown for (from left to right) non-integrative sPLS-DA (applied to each data type separately) and integrative DIABLO models (applied to all data types jointly). Model generalizability varied most between data types, with the proteomic, metabolomic, and especially transcriptomic data resulting in more generalizable models overall, while models built from the flow cytometry and plasma cytokine/chemokine data were less so. An optimal complexity, a point at which the inclusion of additional features did not result in a better ability to generalize to unseen samples, was observed for all models. An integrative DIABLO model composed of 20 features and 2 components generalized best (error rate = 5.76 ± 2.76%).


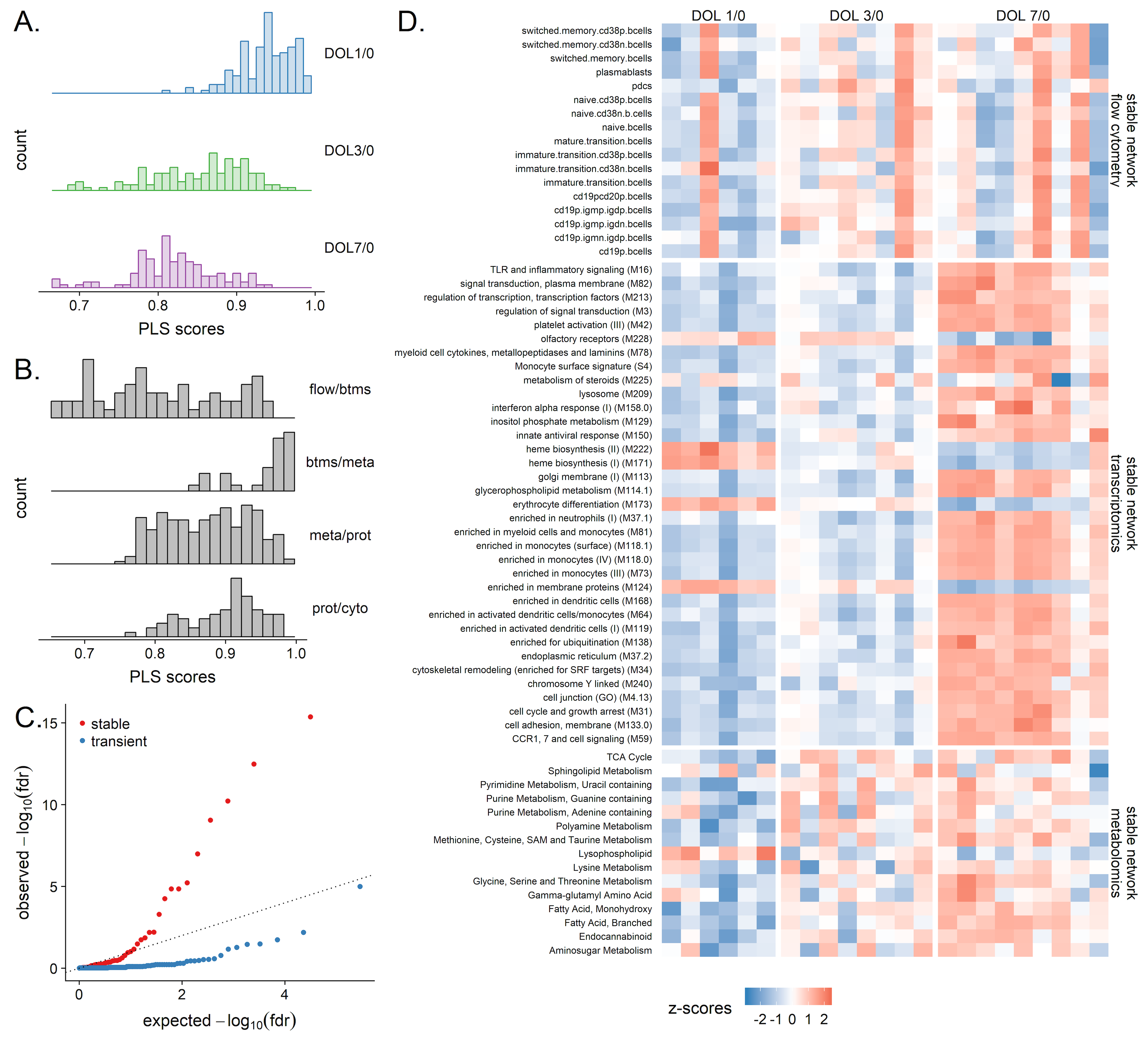


**Supplementary Figure 7.** Multi-scale, multifactorial response networks identified a small set of biological processes consistently associated with each other across all DOLs. Networks were constructed separately at each DOL and stable clusters identified. The partial least squares regression scores, a measure of the strength of association between data types, were highest at DOL 1 and decreased across the first week of life are shown in **A**; the transcriptomic and metabolomic data were most strongly associated as indicated in **B**, followed by the proteomics and cytokine data, metabolomic and proteomic, and flow cytometry and transcriptomic. Association of stable and transient clusters with DOL was assessed using the Correlation-Adjusted Mean RAnk (CAMERA) method. **C** shows the comparison of the distribution of the observed Benjamini-Hochberg false discovery rates to expected quantiles in a Quantile-quantile (QQ) plot. Stable clusters were more strongly associated with DOL compared to transient ones. Finally, a small set of stable clusters were consistently associated at all 3 DOLs, forming a stable sub-network; the features that make up this stable sub-network are visualized in a heatmap in **D**.


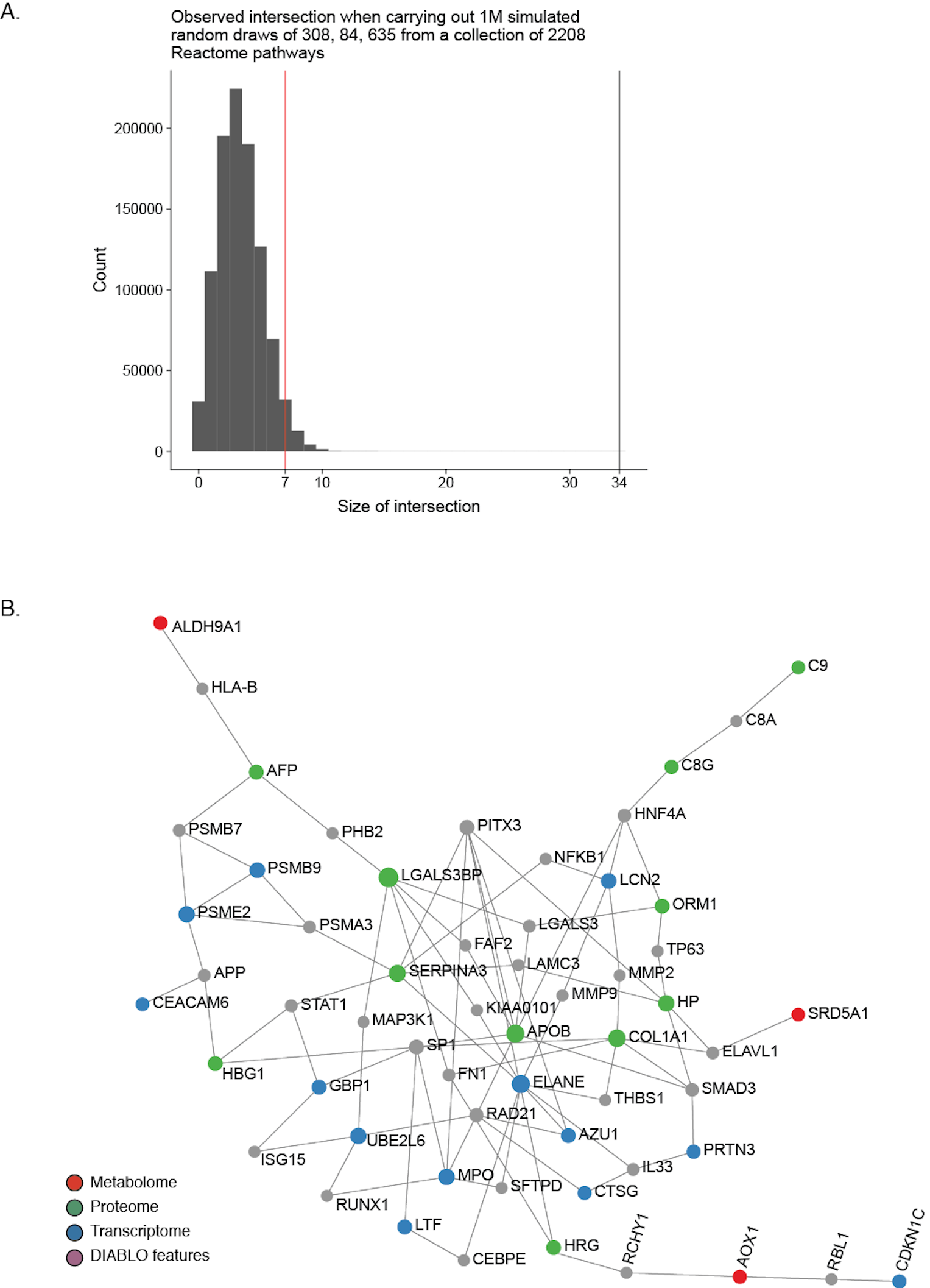


**Supplementary Figure 8. Meta-integration to identify convergence across data integration strategies. A.** Simulation study. Sets of Reactome pathways (2208) were randomly drawn (308 DIABLO, 84 MMRN, 635 NetworkAnalyst), their overlap noted, and the process repeated 1 million times. The results show that overlap as large as that observed in Figure 6A is highly unlikely to have occurred by chance. **B.** *DIABLO*-selected features from components 1 and 2 formed a functional, minimum connected molecular interaction network, containing nodes from the proteome (green), metabolome (red) and transcriptome (blue) as visualized using NetworkAnalyst.

**
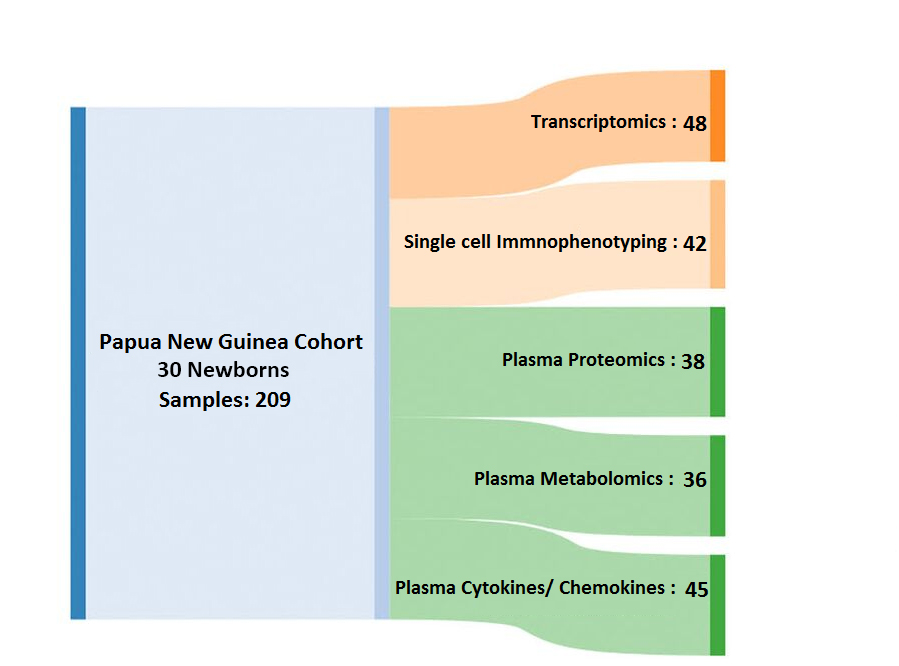
**

**Supplementary Figure 9. Sample flow in the Papua New Guinea cohort.** Diagram displaying sample numbers at each processing step. We collected blood samples from 30 newborns over the first week of life in our Papua New Guinea (PNG) cohort. These were processed according to the diagram in Figure 1. Only subjects for whom we had obtained paired samples (i.e. DOL0 plus either DOL1, 3 or 7), and only those where both samples passed quality controls were subsequently run in the specific OMIC platform. This approach led to the exclusion of 12 samples from transcriptomic analysis, 18 from flow cytometric analysis, 22 from proteomic, 24 from metabolomic and 15 from cytokine/chemokine analysis. In total we processed 209 samples from the Papua New Guinea cohort of newborns.


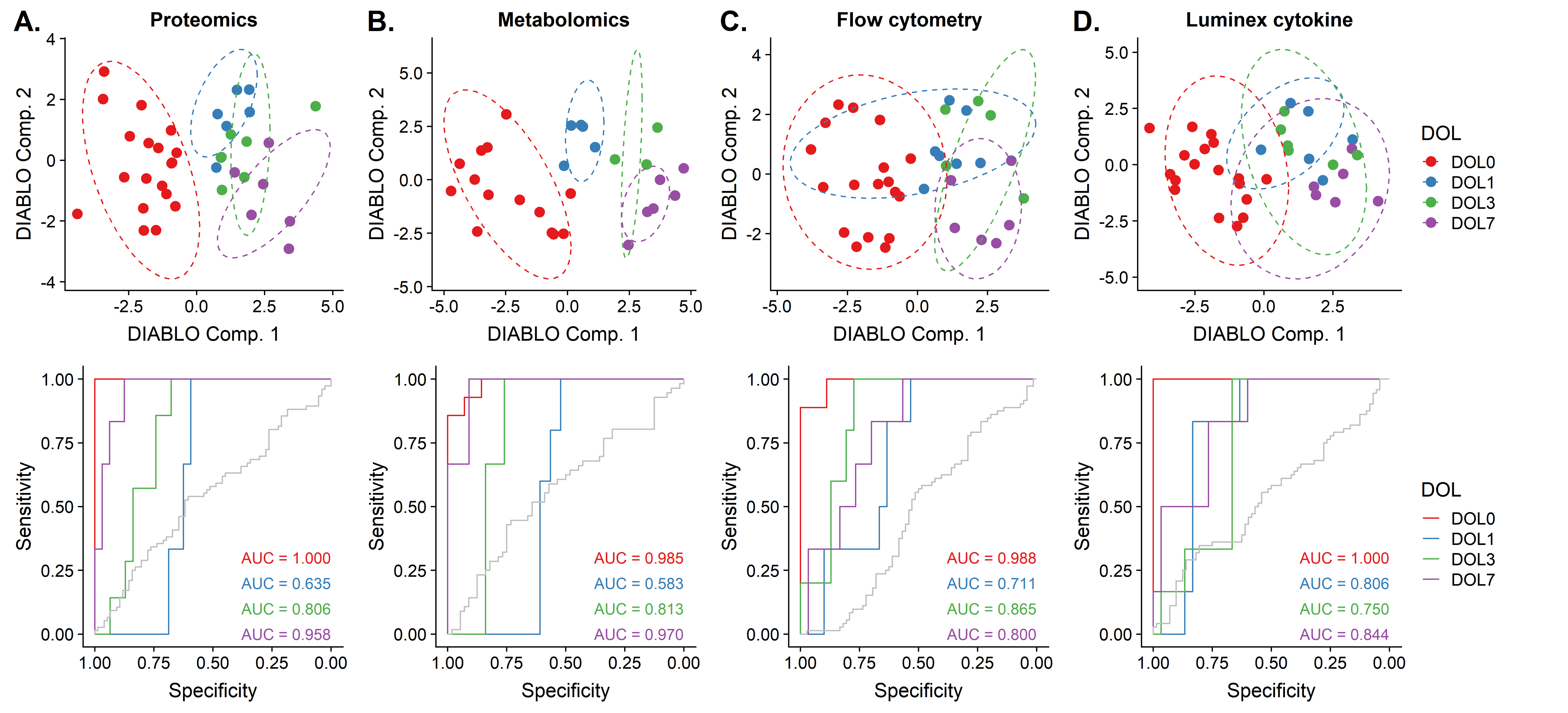


**Supplementary Figure 10.** Independent validation of a robust developmental trajectory in the Papua New Guinea cohort. As shown in Figure 6B and C for transcriptomic data, we also assessed the ability of the multivariate integrative DIABLO model trained on the Gambian newborn data set to classify DOL for **A**. proteomic, **B**. metabolomic, **C**. flow cytometric or **D**. cytokine/chemokine (Luminex) profiles obtained processing new samples collected from newborns in Papua New Guinea. To this end, the DIABLO component scores were computed from the respective omics profiles of samples not used to train and visualized (top panel, colored by DOL). Dashed lines represent the 95% confidence level ellipses for the component scores obtained from the Gambian training data. When visualized in this context, samples from the Papua New Guinea site were found to generally fall within the correct ellipse, demonstrating good agreement between actual DOL and DOL as predicted by our model. This agreement was quantified in the bottom panel using area under the receiver operator characteristics curve (AUROC) analysis comparing DOL 0 (red), 1 (blue), 3 (green), and 7 (purple) vs. all other DOLs. Consistently, DOL0 was most readily differentiated from the other DOLs.

**Supplementary Figure 11.** Sample of gating strategy. See Supplementary Note 1, Section 4.3 for details.

# List of Abbreviations (as they appear in the text)

DOL: Day of Life

SOP: Standard Operating Procedure

RBC: Red Blood Cells

WBC: White Blood cells

FCM: Flow Cytometry

FCS: Flow Cytometry Standard

pDC: Plasmocytoid Dendritic cells

PCA: Principle Component Analysis

DC: Dendritic cells

mDC: Myeloid Dendritic cells

CXCL10: C-X-C motif chemokine 10

GCSF: Granulocyte Colony Stimulating Factor

DEG: Differentially Expressed Genes

MDC: Macrophage Derived Chemokine

IP10: Interferon-gamma induced Protein 10

IFNY: Interferon Gamma

Flt3-L: FMS Like Tyrosine 3 Ligand

CCL5: Chemokine C-C motif ligand 5

TGFalpha: Transforming Growth Factor alpha

IL (17, 10, 6): Interleukin (17, 10, 6)

RIN: RNA Integrity Number

Hb: Hemoglobin

DE: Differentially Expressed

RIG-I: Retinoic Acid Inducible Gene I

FDR: False Discovery Rate

PPI: Protein-Protein Interactions

*DIABLO*: Data Integration Analysis for Biomarker Discovery using a Latent Component method for Omics

*MMRN*: Multi-scale, Multi-Response Network

PLS: Projection to Latent Structures or Partial Least Squares

BTM: Blood Transcriptomic Modules

CCR (1,7): C-C Chemokine Receptor (1,7)

TLR: Toll Like Receptor

DHX:  DEAH-box helicases

PTGS: Prostaglandin Endoperoxide Synthase

PNG: Papua New Guinea

# Supplementary Note 1: MiFlowCyt

1. Experiment Overview
   1. Purpose:

This panel was designed to capture as much information about immune cell populations as possible from a very limited amount of blood.

- 1. Keywords:

Human whole blood, neonatal whole blood, low volume samples, immune cell populations.

- 1. Experiment Variables:

Comparing immune cell populations of neonates over the 1^st^ week of life

- 1. Organization:
     1. Name:

Kollmann Lab, University of British Columbia

- - 1. Address:

950 W 28^th^ Ave, Vancouver, British Columbia, V5Z 4H4 Room A5-175

- 1. Primary Contact:
     1. Name:

Tobias Kollmann MD PhD

- - 1. Address:

UBC, CFRI A5-175. 950 W 28th Ave Vancouver, BC V5Z4H4

- - - 1. Email:

tkollm@mac.com

- - - 1. Tel:

(604) 875-2466

- 1. Date:

Development of the original panel began in January of 2011, and was most recently updated in 2017 (PMID: 28419701). The panel employed here was slightly modified further for the purposes of this particular experiment.

- 1. Conclusions:

This 15-parameter (13 colors; FSC, SSC) whole blood panel captures most immune cell populations, live-dead discrimination, absolute cell numbers as well as relative proportions and reveals dramatic changes over the 1^st^ week of life.

- 1. Quality Control Measures

FMO and single-stain controls.

1. Flow Sample / Specimen Details
   1. Sample / Specimen Material Description
      1. Biological Samples
         1. Biological Sample Description:

Whole blood obtained by peripheral blood venipuncture

- - - 1. Biological Sample Source Description:

Healthy neonates enrolled in study, obtained and processed <4hr from collection

- - - 1. Biological Sample Source Organism Description
         1. Taxonomy:

Kingdom Animalia Subkingdom Metazoa Phylum Chordata Subphylum Vertebrata Superclass Tetrapoda Class Mammalia Subclass Theria Infraclass Eutheria Order Primates Suborder Anthropoidea Family Hominidae Subfamily Homininae Tribe Hominini Genus Homo Subspecies sapiens

- - - - 1. Age:

All peripheral blood samples were collected within the first week of life, either on day of life 0, 1, 3, or 7

- - - - 1. Sex:

Male and Female

- - - - 1. Phenotype:

Healthy (none)

- - - - 1. Genotype:

Not applicable

- - - - 1. Treatment:

Not applicable

- - 1. Environmental Samples:

Not applicable

- - 1. Control Sample Description:

Single stain controls were set up by staining 5 ul of Anti-Mouse Ig CompBeads (BD #552843), Anti-Rat Ig CompBeads (BD #552843), and 5 ul of anti-FBS negative control beads (included with BD #552843) with 3 ul of each antibody used.

- 1. Sample Treatment(s) Description:

*Blood Collection.* Peripheral blood was drawn via sterile venipuncture into 4 ml Vacutainers containing 75 units of sodium-heparin (Becton Dickinson (BD) Biosciences, catalog no. 367871). Blood samples were kept at room temperature and processed within < 4 hrs of the blood draw as described above.

*Sample Processing.* EDTA was added to whole blood within 4 hrs of collection. Cells were stained with fixable variability dye prior to lysis and stored at -80°C in Smart Tube reagents (Smart Tube Inc., San Carlos, California. Upon thawing, samples were washed in staining buffer (PBSAN; 0.5% BSA, 0.1% sodium azide) and stained at room temperature in PBSAN with a cocktail of markers to determine the frequency of cell populations (Supplementary Data 1). Samples were kept in the dark while staining.

- 1. Fluorescence Reagent(s) Description:

See Supplementary Data 1

1. Instrument Details
   1. Instrument Manufacturer:

BD Biosciences

- 1. Instrument Model:

BD LSR II

- 1. Instrument Configuration and Settings:

The BD LSR contained 4 lasers and can detect up to 19 parameters.

See Supplementary Data 2.

1. Data Analysis Details
   1. FCS Data File:

To request raw data please contact **Dr. Tobias Kollmann** at tkollm@mac.com

- - 1. Total Count of Events:

Recorded within individual FCS files were 500,000 events pre-sample

- 1. Compensation Description

Compensation was done in FlowJo using BD CompBeads as single stain controls. A representative compensation matrix is shown Supplementary Data 3.

- 1. Gating (Data Filtering) Description:

**Supplementary Data 1.** Fluorescence Reagents and Descriptions (see Section 2.3).

**Supplementary Data 2.** Instrument Configuration and Settings (see Section 3.3)

**Supplementary Data 3.** Compensation matrix (see section 4.2)

**Supplementary Data 4.** Tabulation of anchor markers used to identify predefined target populations by flow cytometry

**Supplementary Figure 11.** Sample of gating strategy (see section 4.3)

# Supplementary References

1. Li, S. *et al.* Metabolic Phenotypes of Response to Vaccination in Humans. *Cell* **169**, 862-877.e817 (2017).

2. Smith, C.L. *et al.* Identification of a human neonatal immune-metabolic network associated with bacterial infection. *Nature communications* **5**, 4649 (2014).

3. Schroeder, A. *et al.* The RIN: an RNA integrity number for assigning integrity values to RNA measurements. *BMC molecular biology* **7**, 3 (2006).

4. Collins, F.S. & Weissman, S.M. The molecular genetics of human hemoglobin. *Progress in nucleic acid research and molecular biology* **31**, 315-462 (1984).

5. Shin, H. *et al.* Variation in RNA-Seq transcriptome profiles of peripheral whole blood from healthy individuals with and without globin depletion. *PloS one* **9**, e91041 (2014).

6. Beger, R.D. *et al.* Metabolomics enables precision medicine: "A White Paper, Community Perspective". *Metabolomics : Official journal of the Metabolomic Society* **12**, 149 (2016).

7. Spicer, R., Salek, R.M., Moreno, P., Canueto, D. & Steinbeck, C. Navigating freely-available software tools for metabolomics analysis. *Metabolomics : Official journal of the Metabolomic Society* **13**, 106 (2017).

8. Tibshirani, R. Regression Shrinkage and Selection via the Lasso. (1996).

9. Janes, K.A. *et al.* A systems model of signaling identifies a molecular basis set for cytokine-induced apoptosis. *Science* **310**, 1646-1653 (2005).

10. Newman, M.E. & Girvan, M. Finding and evaluating community structure in networks. *Physical review. E, Statistical, nonlinear, and soft matter physics* **69**, 026113 (2004).

11. Le Cao, K.A., Boitard, S. & Besse, P. Sparse PLS discriminant analysis: biologically relevant feature selection and graphical displays for multiclass problems. *BMC bioinformatics* **12**, 253 (2011).

12. Pettengill, M. *et al.* Soluble ecto-5'-nucleotidase (5'-NT), alkaline phosphatase, and adenosine deaminase (ADA1) activities in neonatal blood favor elevated extracellular adenosine. *J Biol Chem* **288**, 27315-27326 (2013).

13. Iacovidou, N. *et al.* Metabolomics applied in neonatology. *Bioanalysis* **6**, 403-410 (2014).

14. Ngo, C.C. & Man, S.M. Mechanisms and functions of guanylate-binding proteins and related interferon-inducible GTPases: Roles in intracellular lysis of pathogens. *Cellular microbiology* **19** (2017).

15. Basler, M., Kirk, C.J. & Groettrup, M. The immunoproteasome in antigen processing and other immunological functions. *Curr Opin Immunol* **25**, 74-80 (2013).

16. Yi, A.K., Chace, J.H., Cowdery, J.S. & Krieg, A.M. IFN-gamma promotes IL-6 and IgM secretion in response to CpG motifs in bacterial DNA and oligodeoxynucleotides. *J Immunol* **156**, 558-564 (1996).

17. Albina, J.E. & Reichner, J.S. Role of nitric oxide in mediation of macrophage cytotoxicity and apoptosis. *Cancer Metastasis Rev* **17**, 39-53 (1998).

18. MacMicking, J.D., Taylor, G.A. & McKinney, J.D. Immune control of tuberculosis by IFN-gamma-inducible LRG-47. *Science* **302**, 654-659 (2003).

19. Lawrence, S.M., Corriden, R. & Nizet, V. Age-Appropriate Functions and Dysfunctions of the Neonatal Neutrophil. *Frontiers in pediatrics* **5**, 23 (2017).

20. Grumach, A.S., Ceccon, M.E., Rutz, R., Fertig, A. & Kirschfink, M. Complement profile in neonates of different gestational ages. *Scandinavian journal of immunology* **79**, 276-281 (2014).

21. Kollmann, T.R., Kampmann, B., Mazmanian, S.K., Marchant, A. & Levy, O. Protecting the Newborn and Young Infant from Infectious Diseases: Lessons from Immune Ontogeny. *Immunity* **46**, 350-363 (2017).

22. Recalcati, S., Locati, M. & Cairo, G. Systemic and cellular consequences of macrophage control of iron metabolism. *Seminars in immunology* **24**, 393-398 (2012).

23. Bullen, J., Griffiths, E., Rogers, H. & Ward, G. Sepsis: the critical role of iron. *Microbes and infection / Institut Pasteur* **2**, 409-415 (2000).

24. Sturgeon, P. Studies of iron requirements in infante and children. I. Normal values for serum iron, copper and free erythrocyte protoporphyrin. *Pediatrics* **13**, 107-125 (1954).

25. Szabo, M. *et al.* Acute postnatal increase of extracellular antioxidant defence of neonates: the role of iron metabolism. *Acta Paediatr* **90**, 1167-1170 (2001).

26. Hay, G. *et al.* Predictors of serum ferritin and serum soluble transferrin receptor in newborns and their associations with iron status during the first 2 y of life. *The American journal of clinical nutrition* **86**, 64-73 (2007).

27. McFall-Ngai, M. *et al.* Animals in a bacterial world, a new imperative for the life sciences. *Proc Natl Acad Sci U S A.* **110**, 3229-3236. doi: 3210.1073/pnas.1218525110. Epub 1218522013 Feb 1218525117. (2013).

28. Burl, S. *et al.* Age-dependent maturation of Toll-like receptor-mediated cytokine responses in Gambian infants. *PloS one* **6**, e18185 (2011).

29. Bennett, L.D., Fox, J.M. & Signoret, N. Mechanisms regulating chemokine receptor activity. *Immunology* **134**, 246-256 (2011).

30. Cohen, P. The TLR and IL-1 signalling network at a glance. *Journal of cell science* **127**, 2383-2390 (2014).

31. Carr, E.J. *et al.* The cellular composition of the human immune system is shaped by age and cohabitation. *Nat Immunol* **17**, 461-468 (2016).

32. Tsang, J.S. *et al.* Global analyses of human immune variation reveal baseline predictors of postvaccination responses. *Cell* **157**, 499-513 (2014).
