# Supplementary material for: Dynamic molecular changes during the first week of human life follow a robust developmental trajectory
Source: Nat Commun. 2019 Mar 12;10:1092. doi: 10.1038/s41467-019-08794-x (PMC6414553; doi:10.1038/s41467-019-08794-x)
Supplement: Supplementary file 2 — Description of Additional Supplementary Files [file 41467_2019_8794_MOESM2_ESM.docx]

**Description of Additional Supplementary Files**

File Name: Supplementary Data 1

Description: Fluorescence Reagents and Descriptions (see Supplementary Information section 2.3)

File Name: Supplementary Data 2

Description: Instrument Configuration and Settings (see Supplementary Information section 3.3)

File Name: Supplementary Data 3

Description: Compensation matrix (see Supplementary Information section 4.2)

File Name: Supplementary Data 4

Description: Tabulation of anchor markers used to identify predefined target populations by flow cytometry

File Name: Supplementary Data 5

Description: List of all differentially expressed genes, pathway enrichment and statistics

File Name: Supplementary Data 6

Description: List of differentially abundant plasma proteins and their respective pathways

File Name: Supplementary Data 7

Description: List of differentially abundant plasma metabolites and their respective pathways

File Name: Supplementary Data 8

Description: PPI-based integration across different OMICS data sets

File Name: Supplementary Data 9

Description: DIABLO based integration across different OMICS data sets

File Name: Supplementary Data 10

Description: MMRN based integration across different OMICS data sets

File Name: Supplementary Data 11

Description: Pathway enrichment similarities across PPI, DIABLO and MMRN based integration of different OMICS data sets

File Name: Supplementary Data 12

Description: Meta-integration of PPI, DIABLO and MMRN based OMICS integration
